# Acidic transformation of nordiazepam can affect recovery estimate during trace analysis of diazepam and nordiazepam in environmental water samples by liquid chromatography–tandem mass spectrometry

**DOI:** 10.1007/s00216-019-01870-7

**Published:** 2019-05-18

**Authors:** Victoria K. H. Barclay, Niklas L. Tyrefors, I. Monika Johansson, Curt E. Pettersson

**Affiliations:** Department of Medicinal Chemistry, Analytical Pharmaceutical Chemistry, Uppsala University, BMC Box 574, SE-751 23 Uppsala, Sweden

**Keywords:** *N*-desmethyldiazepam, Nordazepam, Wastewater, Hydrolysis, Stability, LC-MS/MS

## Abstract

In this study, a special interest was focused on the stability of diazepam and nordiazepam in aqueous samples at acidic and neutral pH. The aim of the study was to isolate and illustrate one of the many possible sources of error that can be encountered when developing and validating analytical methods. This can be of particular importance when developing multi-analyte methods where there is limited time to scrutinize the behavior of each analyte. A method was developed for the analysis of the benzodiazepines diazepam and nordiazepam in treated wastewater. The samples were extracted by solid phase extraction, using SPEC C18AR cartridges, and analyzed by the use of liquid chromatography, with a C18 stationary phase, coupled to tandem mass spectrometry. Environmental water samples are often acidified during storage to reduce the microbial degradation of the target compounds and to preserve the sample. In some cases, the samples are acidified before extraction. In this study, it was found that a chemical equilibrium between nordiazepam and a transformation product could cause inaccurately high extraction recovery values when the samples were stored at low sample pH. The stability of nordiazepam was shown to be low at pH 3. Within 12 days, 20% of the initial concentration of nordiazepam was transformed. Interestingly, the transformed nordiazepam was shown to be regenerated and reformed to nordiazepam during sample handling. At a sample pH of 7, diazepam and nordiazepam were stable for 12 days. It was concluded that great care must be taken when acidifying water samples containing nordiazepam during storage or extraction. The storage and the extraction should be conducted at neutral pH if no internal standard is used to compensate for degradation and conversion of nordiazepam. The developed method was validated in treated wastewater and applied for the quantification of diazepam and nordiazepam in treated wastewater samples.

## Introduction

Diazepam is a psychotropic active pharmaceutical ingredient (API) belonging to the group of benzodiazepines. In a clinical context, diazepam is mainly used to treat the symptoms of anxiety and to reduce increased muscle tone. Diazepam is metabolized in humans to form nordiazepam (also known as *N*-desmethyldiazepam and nordazepam) which is a pharmacologically active metabolite that also is formed from a number of other benzodiazepines. Nordiazepam is further metabolized to the active metabolite oxazepam. Oxazepam is clinically used as an anxiolytic and hypnotic active pharmaceutical ingredient [1]. Benzodiazepines are nowadays one of the most commonly prescribed groups of pharmaceuticals due to their broad range of therapeutic effects [2]. However, benzodiazepines are also often abused. Drugs of abuse are a group of emerging environmental contaminants that have recently received a large amount of attention [3–5].

Today, it is known that many APIs and their metabolites are discharged into the aquatic environment by wastewater treatment plants [6–10]. Diazepam is one of the APIs that has been detected in sewage treatment plant influents [11] and effluents [8, 12], and in river water [12, 13]. The active metabolite nordiazepam has also been detected in wastewater treatment plant influents [12] and effluents [12, 14, 15], as well as in river water [12, 15]. In the aquatic environment, diazepam can be classified as potentially harmful to aquatic organisms [16].

For these reasons, diazepam and/or nordiazepam are often included in the so-called multi-residue methods developed for the monitoring and routine analysis of pharmaceutical residues and their metabolites in the aquatic environment [12, 17, 18]. Up to 105 different analytes can be included in one single method [19] and many multi-residue analytical methods are published. Therefore it is of importance to focus on some of the many analytical details that might be overlooked when 50 − ≥ 100 analytes are included in one single method [20]. The samples are often extracted by the use of generic solid phase extraction (SPE) methods and further analyzed by mass spectrometry. Different types of SPE sorbents, such as Oasis MCX at low sample pH [11, 12, 14, 15] or Oasis HLB at neutral sample pH [13] have been employed to extract benzodiazepines and their metabolites from environmental matrices. These two polymeric sorbents have frequently been used for multi-residue analysis where the target compounds have widely different physicochemical characteristics. Octadecylsilane (C18) SPE cartridges have also been applied [21] and have commonly been used for the extraction of benzodiazepines in biological samples [22].

Water samples are often acidified after collection [11, 12, 15, 23] to increase the stability or to adjust sample pH (for basic analytes) before SPE by the use of cationic exchange sorbents. Acidification of environmental water samples is known to prevent bacterial activity and therefore preserve the samples [24]. Moreover, water samples are often extracted within 30 h of sampling [7, 12] or stored for no more than 1 week [23] or 14 days [21]. In a recent publication [3], the stability of more than 60 compounds in wastewater samples was studied at pH 2 and 7, and at 2 and 19 °C. However, the stability of the analytes in the samples is often overlooked [25]. It is known from the literature that some benzodiazepines undergo hydrolysis under acidic conditions [26] and that nordiazepam forms *N*-(2-benzoyl-4-chlorophenyl)-2-aminoacetamide at low sample pH [27].

The aim of this study was to investigate the stability and solid phase extraction procedure for two analytes that are often included in multi-analyte methods, i.e., diazepam and nordiazepam. A special interest was focused on the effect of chemical equilibrium between nordiazepam and a transformation product on the extraction recovery determinations. In the present study, (I) the stability of diazepam and nordiazepam was studied in an acidic and neutral water sample to simulate the storage conditions in acidified or neutral wastewater, (II) a solid phase extraction method for the target analytes was developed, and (III) the extraction recoveries were determined for diazepam and nordiazepam in an environmental sample that had been simulated to be stored under acidic conditions. The overall aim of this study was to investigate and highlight two observations (i.e., the extraction recoveries and stability) that need careful analytical assessments but actually might be overlooked when multi-analyte methods are developed.

## Materials and methods

### Chemicals and stock solutions

**Fig. 1:**
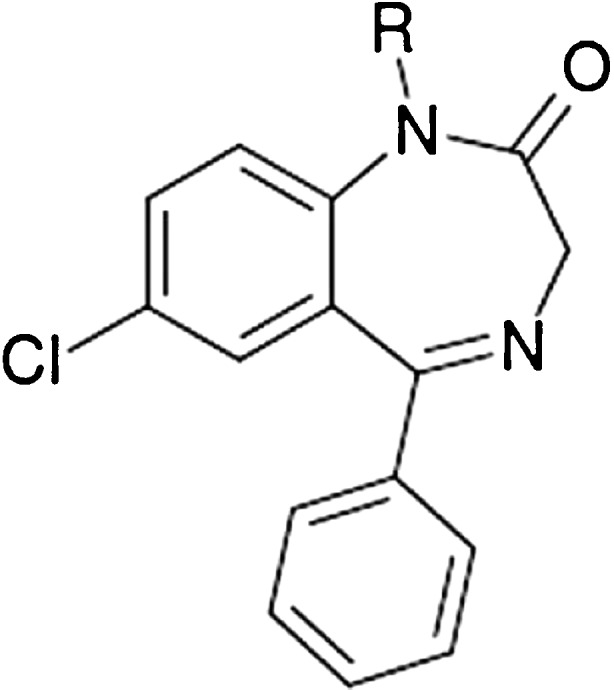
The chemical structures of diazepam and nordiazepam. Diazepam (R = CH_3_) and nordiazepam (R = H)

Diazepam (C_16_H_13_ClN_2_O, 284.74 g mol^−1^) and nordiazepam (C_15_H_11_ClN_2_O, 270.71 g mol^−1^) (Fig. 1), diazepam-d_5_ (isotopic purity; d_0_ 0.00%) and nordiazepam-d_5_ (isotopic purity; d_0_ 0.02%) were obtained as stock solutions in methanol (all with the chemical purity of 99%) from Cerilliant (Round Rock, TX, USA). Formic acid (> 99%, pro analysi) was purchased from Acros Organics (Morris Plains, NJ, USA). Ortho-phosphoric acid (85%, pro analysi) and sodium hydroxide (≥ 99%, pro analysi) came from Merck (Darmstadt, Germany). Methanol (HPLC grade) and acetonitrile (HPLC grade) were from Fisher Scientific UK Limited (Loughborough, UK). A Milli-Q Academic water purification system (Billerica, MA, USA) with a Millipak-40 filter unit (0.22 μm, Billerica, MA, USA) was used to generate purified water.

The stock solutions of diazepam, diazepam-d_5_, nordiazepam, and nordiazepam-d_5_ were stored at − 28 °C in the dark and diluted to working solutions with a mixture of 10 mM formic acid in purified water/methanol (1/1, *v*/*v*) or 5 mM formic acid in purified water/acetonitrile (90/10, *v*/*v*). The working solutions were stored at 4 °C in the dark. Phosphate buffer (pH 7.0, 10 mM) was prepared from stock solutions of phosphoric acid (1 M) and sodium hydroxide (1 M).

### Experimental

#### Sample preparation and solid phase extraction

Grab samples of effluent (treated) wastewater were collected in amber glass bottles (2.5 L) from the wastewater treatment system Kungsängsverket (Uppsala, Sweden). At Kungsängsverket, the water undergoes physical, biological, and chemical treatment. Surface water was collected, as grab samples in 2.5-L amber glass bottles, from the River Fyris, Uppsala. The sampling point was located 3 km upstream from the wastewater treatment plant. The samples were transported for about 20 min and thereafter filtered through glass fiber prefilters from Millipore (0.7 μm, Billerica, MA, USA) or centrifuged at 2000 rpm for 10 min. The pH of the treated wastewater samples were 7.4 and 7.1 and the pH of the surface water samples were 7.7 and 7.3. The filtrate or supernatant was, if necessary, adjusted to pH 7.0 with 1 M H_3_PO_4_ and stored at 2 °C in the dark until the solid phase extraction, which was performed within 6 h of sampling. The pH was measured by the use of a “pH Meter 744” with a Metrohm electrode (Metrohm, Herisau, Switzerland). SPEC C18AR columns (35 mg/10 mL) from Agilent Technologies (Santa Clara, CA, USA) were used to extract the samples of 75.0 mL wastewater or surface water. Each cartridge was conditioned with methanol (5.0 mL) and purified water (5.0 mL) and equilibrated with 10 mM phosphate buffer pH 7.0 (5.0 mL). The extractions were performed by the use of a Sep-Pak vacuum manifold (Waters, Milford, MA, USA). The water samples were applied by 70 mL Isolute column reservoirs (Biotage, Uppsala, Sweden), which were connected to the extraction columns by the use of column adaptors (Phenomenex, Torrance, CA, USA). The sample application flow rate was approximately 1.5 mL min^−1^ and the columns were washed with 2.0 mL of 10 mM phosphate buffer at pH 7.0/acetonitrile (95/5, *v*/*v*) without subsequent drying. The analytes were eluted with methanol (4.0 mL) and the extracts were evaporated until dry at 60 °C under a gentle stream of nitrogen. The dry residues were reconstituted in 250 μL of 5 mM formic acid in purified water/acetonitrile (90/10, *v*/*v*) or in 250 μL of 10 mM phosphate buffer pH 7.0. The reconstituted extracts were injected into the LC system (“The chromatographic systems”).

During the development of the solid phase extraction method, the extraction recoveries were determined for the analytes or the isotope-labeled compounds in different sample matrices, i.e., phosphate buffer, surface water, or in treated wastewater according to the methodology described in “Method validation.”

The laboratory glassware used in this study was washed with detergent (Neodisher FLA, Chemische Fabrik Dr. Weigert GmbH & Co. KG, Hamburg, Germany) in a laboratory glassware washer (Miele G7783, Miele, Gutersloh, Germany). Two prewashing cycles and one washing cycle (maximum temperature of 85 °C) were included in the washing sequence as well as four rinse cycles and one drying stage.

#### The chromatographic systems

Two LC systems were used and in both systems a YMC Pack Pro C18 column, 50 × 2.1 mm (5 μm) (YMC Europe GmbH Dinslaken, Germany) was connected to a security guard cartridge holder with a C8 security guard cartridge (4 × 2 mm) from Phenomenex (Skandinaviska Genetec AB, Västra Frölunda, Sweden). The mobile phase A consisted of 5 mM formic acid in purified water and mobile phase B of 5 mM formic acid in acetonitrile. The flow rate was set to 0.25 mL min^−1^ in both systems. Further information on the LC systems is given below; the mass spectrometry detection settings are given in “Mass spectrometry detection.”

The Agilent 1100 HPLC system (equipped with degasser, binary pump, auto sampler, and UV detector) from Agilent Technologies Inc. (Palo Alto, CA, USA) was used for isocratic separations. The mobile phase used was 40/60 (*v*/*v*) of A/B in the isocratic system. This LC system was also used for LC-UV determinations of diazepam and nordiazepam at 238 nm. The injected volume was 20 μL and the separations were performed at ambient temperature.

The Acquity UPLC system (Waters Corporation, Milford, MA, USA) was used for gradient elution. The gradient for mobile phase B was as follows: initial—30%, 2 min—65%, 2.10 min—100%, 2.80 min—100%, and 2.81 min—30%. The separations were carried out at an ambient temperature and the injected volume was set to 20 μL.

#### Mass spectrometry detection

A Quattro Micro mass spectrometer (Waters Corporation, Milford, MA, USA) was connected to the Agilent 1100 LC system (“The chromatographic systems”) and a Quattro II (Micromass UK Ltd., Manchester, UK) was used as a detector to the Acquity UPLC system (“The chromatographic systems”). Both mass spectrometers, equipped with electrospray ionization interfaces in Z spray configuration, were used for method development and for the analysis of diazepam and nordiazepam in the water samples. The MS/MS parameters were optimized by the direct infusion of diazepam, nordiazepam, diazepam-d_5_, or nordiazepam-d_5_ into the mass spectrometer. The analytes were detected in positive ion mode and the used SRM channels are given in Table 1. Two fragment ions (product ions 1 and 2) each were selected for diazepam and nordiazepam for the identification of the “naturally” occurring compounds in the environmental samples. The dwell time was set to 0.30 s. The capillary and cone voltages were set to 3.5 kV and 25 V, respectively. The collision cell was filled with argon at a pressure of 3.0 × 10^−3^ mBar and the collision energy used was 27 V. The source block and desolvation temperatures were 80 and 375 °C, respectively. Nitrogen was used as desolvation and cone gas at a flow rate of 10 × 10^3^ and 1.7 × 10^3^ mL min^−1^, respectively. Nitrogen was also used as the nebulizing gas. For data acquisition and peak integration, the MassLynx software program, version 4.0 or 4.1 (Waters Corporation, Milford, MA, USA), was used.

**Table 1:** SRM transitions and retention times for the analytes and the isotope-labeled compounds. The SRM transitions and product ion ratios (*n* = 10) used for diazepam and nordiazepam in the tandem quadrupole mass spectrometer. Retention times (*n* = 6) in treated wastewater in the gradient elution LC system are given. For experimental details see “The chromatographic systems” and “Mass spectrometry detection”

Compound	Precursor ion	Product ion 1	Product ion 2	Ratio ion 2 / ion 1		Treated wastewater	
	*m*/*z*	*m*/*z*	*m*/*z*	Ratio	RSD %	*t*_R_ [min]	RSD %
Diazepam	285	193	154	1.05	5.4	2.8	0.29
Diazepam-d_5_	290	198	–			2.8	0.29
Nordiazepam	271	208	140	1.43	7.6	2.4	0.34
Nordiazepam-d_5_	276	213	165 and 140^I)^			2.4	0.35

^I)^Additional ions monitored in “Regeneration of nordiazepam during sample preparation”

#### Stability studies of diazepam and nordiazepam in acidic and neutral aqueous solutions

Working standards of 1.0 μM diazepam and 1.0 μM nordiazepam were prepared in 5 mM formic acid in purified water (pH 3.1)/acetonitrile (90/10, *v*/*v*) or in 10 mM phosphate buffer pH 7.0. These working standards were stored for 12 days at room temperature or at 4 °C in glass bottles covered with aluminum foil. The standards were injected (*n* = 3) into the LC-UV system on the day of preparation and furthermore on eight occasions until day 12. At day 12, 250 μL of the samples were added to 75 mL phosphate buffer pH 7.0 and extracted as described in “Sample preparation and solid phase extraction.” The dry residues of the SPE extracts were reconstituted in 250 μL 5 mM formic acid in purified water/acetonitrile (90/10, *v*/*v*) and immediately injected into the LC-UV system. Since the concentration of standards was high and the matrix was pure, the samples were detected with LC-UV to reduce the variability in the response that is often obtained with ESI-MS when no internal standard is used for compensation.

Nordiazepam and the transformation product of nordiazepam were analyzed in the stored solution of 1.0 μM nordiazepam in 5 mM formic acid in purified water/acetonitrile (90/10, *v*/*v*) by the Quattro Micro mass spectrometer (“Mass spectrometry detection”). The compounds were separated by an LC gradient programmed as follows for mobile phase B: initial—5%, 5 min—70%, 5.5 min—95%, 6.5 min—95%, and 7 min—5%. Mobile phase A was 5 mM formic acid in purified water and mobile phase B was acetonitrile. The flow rate was 0.25 mL min^−1^ and the injected volume was 5 μL. The transformation product of nordiazepam was detected in MS scan mode and was thereafter analyzed together with nordiazepam in selected ion monitoring (SIM) mode. The selected ions in the first quadrupole were *m*/*z* 289 and 271 for the transformed product and nordiazepam, respectively. The stored solution of nordiazepam was injected (*n* = 2) in SIM mode.

To investigate the effect of evaporation on the chemical equilibrium of nordiazepam and the transformation product, 250 μL of the stored solution of nordiazepam was mixed with 4 mL methanol to simulate the SPE extracts (*n* = 2). One blank mixture of 4 mL methanol and 250 μL 5 mM formic acid in purified water/acetonitrile (90/10, *v*/*v*) was also made. The methanol mixtures were evaporated to dryness at 60 °C under a gentle stream of nitrogen and reconstituted in 5 mM formic acid in purified water/acetonitrile (90/10, *v*/*v*) and injected (*n* = 2) into the LC-MS system operating in SIM mode.

#### Method validation

The method was validated, using L-MS/MS, in treated wastewater from the wastewater treatment system Kungsängsverket. The *accuracy* of the method was assessed by determining the solid phase extraction *recoveries* at two concentrations [28, 29], one low (50 pM) and one high (250 pM) concentration for diazepam-d_5_ and nordiazepam-d_5_ in treated wastewater, pH 7.0. The *extraction recoveries* and *matrix effect* (ME) were determined according to a protocol originating in pharmaceutical bioanalysis, as published by Matuszewski et al. [30]. Three sets of samples were prepared as follows: set A was prepared by spiking the treated wastewater before extraction with the isotope-labeled compounds. Set B was prepared by extracting the water samples (un-spiked) and reconstituting the dried extracts with a working solution containing the target analytes dissolved in either 5 mM formic acid in purified water/acetonitrile (90/10, *v*/*v*) or in 10 mM phosphate buffer pH 7.0. Set C was a working solution of the target analytes dissolved in either 5 mM formic acid in purified water/acetonitrile (90/10, *v*/*v*) or in 10 mM phosphate buffer pH 7.0. The *relative* extraction recovery (%) was determined by dividing the chromatographic peak areas achieved from set A by the peak areas achieved from set B. The *absolute recovery* (%) was determined by dividing the peak areas from set A by the ones obtained from set C. The *matrix effect* was obtained by dividing the peak areas from set B by the peak areas from set C.

The *precision*, given as RSD (%) of the retention times, of the chromatographic systems was determined by the injection of extracted wastewater samples (*n* = 6). The *linearity* (expressed as the correlation coefficient, *R*^2^) was determined by conducting calibration curves for diazepam and nordiazepam in treated wastewater samples. The wastewater samples were spiked with diazepam and nordiazepam to four concentrations: zero (non-spiked), 15, 30, and 60 pM and with diazepam-d_5_ and nordiazepam-d_5_ to a concentration of 30 pM. The samples were thereafter extracted and the calibration curves were constructed by plotting the ratio of the peak area of diazepam or nordiazepam to the peak area of the isotope-labeled internal standard (*y*-axis) against the added concentration of diazepam or nordiazepam (*x*-axis). Double injections of each standard were performed.

Possible *cross-contamination*, during sample handling and SPE, and *carryover* in the LC-MS/MS system were determined to assess the risk of sample and instrumental contamination. The possibility of cross-contamination was determined by extracting blank samples of 75 mL 10 mM phosphate buffer (pH 7.0) in parallel with the wastewater samples during quantification. The extracted blank samples were injected in the LC system and the SRM channels were monitored for diazepam and nordiazepam giving peaks with a signal-to-noise ratio ≥ 3. The possibility of carryover in the LC-MS/MS system was studied by injecting 20 μL of purified water at regular intervals in between the water samples and after the injection of standards with a high concentration of the analytes.

The *limit of quantification* (LOQ) and *limit of detection* (LOD) were determined for diazepam-d_5_ and nordiazepam-d_5_ in treated wastewater samples. Samples of 75 mL wastewater were spiked with the isotope-labeled standards to a concentration of 50 pM and extracted as described in “Sample preparation and solid phase extraction.” The LOQ and LOD were defined as the concentrations of diazepam-d_5_ or nordiazepam-d_5_ giving a peak height ten or three times as high as the average peak to peak amplitude of the background noise, i.e., signal-to-noise ratios of ten or three, respectively. Furthermore, the LOQ can also be defined as the concentration of the analyte giving a reproducible and defined peak area with a RSD of 20% [31]. The reconstituted extracts were diluted with 10 mM phosphate buffer pH 7.0 to concentrations of diazepam-d_5_ and nordiazepam-d_5_ corresponding to those of LOQ and LOD. The precision (RSD %, *n* = 3) was determined for the LOQ.

#### Quantification of diazepam and nordiazepam in treated wastewater samples

Diazepam and nordiazepam were quantified in treated wastewater samples by the standard addition method. The centrifuged wastewater samples were divided into eight samples with a volume of 75 mL each. Diazepam-d_5_ and nordiazepam-d_5_ were added, as internal standards, to all samples to a concentration of 30 pM. The samples were further spiked with diazepam and nordiazepam to the following concentrations: zero (no addition), 15, 30, and 60 pM. Duplicates of each concentration were prepared. The calibration curves were constructed by plotting the ratio of the peak area of diazepam or nordiazepam to the peak area of the isotope-labeled internal standard against the added concentration of diazepam or nordiazepam. The linear regression equations were calculated in Excel and the concentrations of diazepam and nordiazepam in the treated wastewater samples were determined as the absolute value of the intercept at the *x*-axis (where *y* = 0) of the calibration curve.

## Results and discussion

### The liquid chromatography–tandem quadrupole mass spectrometry systems

The developed isocratic as well as the gradient elution systems gave good peak resolution (*R*_s_ ≥ 3.1) for diazepam and nordiazepam. In the isocratic system, the retention times were 2.6 min (0.54 RSD %) and 4.1 min (0.27 RSD %) for standards of nordiazepam-d_5_ and diazepam-d_5_, respectively. In the surface water matrix, the retention times were the same as for the standards and the RSD values were 0.62% and 0.30% for nordiazepam-d_5_ and diazepam-d_5_, respectively. The retention times for the working standards in the gradient system were 2.4 min (0.90 RSD %) and 2.9 min (0.70 RSD %) for nordiazepam and diazepam, respectively. The precision of the retention times for the compounds in treated wastewater are given in Table 1, where the RSD values were ≤ 0.35%.

Diazepam and nordiazepam were identified in the treated wastewater samples by their characteristic MS/MS transitions and retention times (Table 1). One precursor ion and two fragment ions were monitored for each compound. For the identification of “naturally” occurring diazepam and nordiazepam, the ratios of the peak areas obtained from the two fragment ions were compared with those of the standards (Table 1). Also, the retention times for the target compounds were compared to the retention times for diazepam-d_5_ and nordiazepam-d_5_ in the same chromatographic run.

### Development of the solid phase extraction method

#### The stability of diazepam and nordiazepam in acidic and neutral aqueous samples

**Fig. 2:**
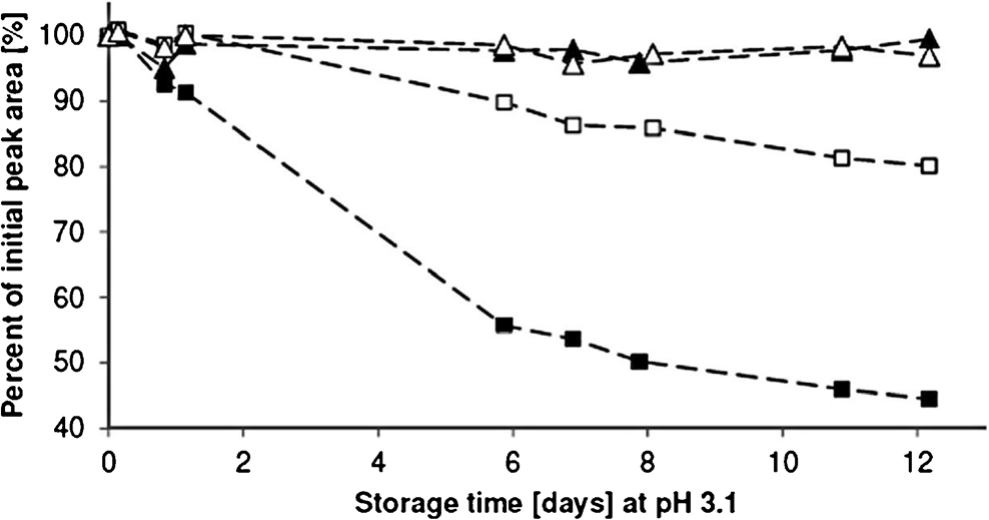
The stability of diazepam and nordiazepam in 5 mM formic acid in purified water, pH 3. Diazepam stored at 4 °C (white triangle) and in room temperature (black triangle). Nordiazepam stored at 4 °C (white square) and in room temperature (black square). Experimental details are described in “Stability studies of diazepam and nordiazepam in acidic and neutral aqueous solutions”

Collected environmental water samples are commonly stored at acidic pH prior to analysis of emerging contaminants [25]. In the present study, the stability of diazepam and nordiazepam were studied when stored as working standards (at pH 3.1 and pH 7.0). It was found that nordiazepam was extensively degraded when stored in the acidic solution at room temperature. Within 12 days, 56% of nordiazepam was degraded (Fig. 2). The degradation was not as extensive when the working solution was stored at 4 °C; after 12 days, 20% of the initial concentration of nordiazepam had degraded. On the other hand, diazepam was found to be stable at pH 3.1 when stored at room temperature and at 4 °C (Fig. 2). Within the 12 days, only 0.53% and 3.1% of diazepam were degraded at room temperature and at 4 °C, respectively. Both compounds were shown to be stable for 12 days at neutral pH. At day 12, the responses for diazepam and nordiazepam (when stored at 4 °C and room temperature) were 101–103% of the initial responses. The RSD values (*n* = 3) for the determined concentration at different time points were ≤ 5.1%. The reaction was initially discovered in wastewater samples but the experiments were performed in prepared solutions to simplify the conditions and reduce the possibility of other reactions/effects in the very complex matrixes of wastewater.

Our results show that nordiazepam is instable at pH 3, these results are in accordance with results in the literature which show that nordiazepam undergoes hydrolysis in acidic solutions. Archontaki et al. [27] found that nordiazepam was hydrolyzed in acidic aqueous solutions and that the first step in the degradation was reversible. However, the findings in the present study may seem contrary to the results from a recent study where it was found that nordiazepam (and diazepam) were more stable at pH 2 than at pH 7 [3]. The difference is actually expected when considering the different strategies for recovery determination. Since the transformation of nordiazepam is reversed during evaporation and heating, there might not be any practical implication for routine use of the method. It is only when the recovery is estimated according to the recommendations by Matuszewski et al. [30]in combination with the unstability of nordiazepam at low pH that we get a loss of the compound and thus an apparent high recovery.

#### The solid phase extraction recoveries for simulated environmental water samples, stored at low sample pH

The solid phase extraction recoveries and the matrix effect were determined in treated water samples by LC-MS using the approach suggested by Matuszewski et al. [30], details are given in “Method validation”. The extraction recoveries were determined for the isotope-labeled standards, diazepam-d_5_ and nordiazepam-d_5_, as labeled compounds are not expected to be found in the environmental matrices [6]. In the present study, the working standards of diazepam-d_5_ and nordiazepam-d_5_ were stored in 5 mM formic acid in purified water pH 3.1/acetonitrile (90/10, *v*/*v*), for no longer than 1 week at 8 °C, and were used to prepare sets A–C.

The relative extraction recoveries, determined at one low and one high concentration, are given in Table 2 (the extraction method is given in “Sample preparation and solid phase extraction”). The extraction recoveries were higher for nordiazepam-d_5_ than for diazepam-d_5_. For nordiazepam-d_5_, the relative extraction recoveries were 114 ± 8.1% and 117 ± 21% at the low and high concentration, respectively. The RSD values obtained for diazepam-d_5_ were 6.0% and 24% for the low and high concentration, respectively (Table 2). High values of RSD (≥ 18%) for the solid phase extraction procedure are sometimes obtained at trace-level determinations in complex matrices [12, 32]. Furthermore, high extraction recoveries (≥ 100%) for nordiazepam in environmental water samples have been reported in the literature previously [12, 13, 32, 33]. As will be discussed below, the high recoveries could be correlated to a chemical equilibrium between nordiazepam and a transformation product.

**Table 2:** The extraction recoveries and matrix effects for diazepam-d_5_ and nordiazepam-d_5_ in surface water. The relative recoveries (%) determined at one low and one high concentration. Absolute recoveries (%) and the matrix effects (%) determined at the high concentration. For experimental details see “Sample preparation and solid phase extraction,” “The chromatographic systems,” and “Mass spectrometry detection”

Compound	Relative recovery^I)^ ± RSD %	Relative recovery^II)^ ± RSD %	Absolute recovery^II)^ ± RSD %	Matrix effect^II)^ ± RSD %
Diazepam-d_5_	103 ± 6.0	87 ± 24	109 ± 24	126 ± 4.0
Nordiazepam-d_5_	114 ± 8.1	117 ± 21	139 ± 21	119 ± 3.0

^I)^50 pM, *n* = 5

^II)^250 pM, *n* = 3

The absolute extraction recovery for nordiazepam-d_5_ was determined to 139 ± 21% and the matrix effect to 119 ± 3.0%. The high absolute extraction recovery for nordiazepam-d_5_ can partly be explained by the fact that nordiazepam-d_5_ was subjected to ion enhancement in the MS interface. However, we showed that the high extraction recoveries (> 100% for the relative extraction, as discussed above) were not only caused by matrix effects in the ESI source. This was determined by the relative extraction recoveries obtained according to Matuszewski et al. [30] where the extracted and the non-extracted samples were injected into the LC-MS/MS system dissolved in the same matrix. In addition, to further verify that the high extraction recoveries were not correlated to any process in the interface of the mass spectrometer, the extraction recoveries for diazepam-d_5_ and nordiazepam-d_5_ were determined by the use of a second detection technique, LC-UV. The recovery for one extracted sample of nordiazepam-d_5_ in phosphate buffer (pH 7.0) analyzed with both LC-MS/MS and LC-UV was determined to be 159 and 153% (*n* = 2), respectively. We concluded that the high extraction recoveries for nordiazepam were not attributed, to a great extent, to any process in the mass spectrometer.

In conclusion, even though the obtained extraction recoveries and the RSD values were high, they would probably appear as reasonable as the concentrations of the target compounds were low (50 and 250 pM) and because the compounds were extracted from a complex matrix. In this study, we wanted to demonstrate that these high recoveries obtained for nordiazepam could be correlated to a chemical equilibrium between nordiazepam and a transformation product found by Archontaki et al. [27].

#### Regeneration of nordiazepam during sample preparation

When stored solutions of diazepam and nordiazepam (pH 3.1, at room temperature, “The stability of diazepam and nordiazepam in acidic and neutral aqueous samples”) were used to spike the phosphate buffer and then subjected to solid phase extraction, the responses obtained from the reconstituted extracts of nordiazepam were larger compared to the responses obtained from the un-extracted stored solutions. By the solid phase extraction, the peak area of nordiazepam had increased from 26 area counts (2.7 RSD %, *n* = 3) to 45 area counts (14.6 RSD %, *n* = 3).

In order to verify that nordiazepam was regenerated during the solid phase extraction, one stored sample of nordiazepam-d_5_ (giving a peak area of 1470 for the fragment ion of *m*/*z* 213) and one processed sample of nordiazepam-d_5_ (with a peak area of 1790) were injected into the LC-MS/MS system. In addition to the SRM channel of nordiazepam-d_5_ (276 → 213), two additional SRM channels were acquired, Table 1. The stored samples of nordiazepam-d_5_ were injected (*n* = 6) and the ratios of the SRM transitions were determined to be 1.4 (3.8 RSD %, fragment ion ratio of *m*/*z* (276 → 213)/(276 → 165) and 1.0 (3.5 RSD %, fragment ion ratio of *m*/*z* (276 → 213)/(276 → 140). For the processed sample of nordiazepam-d_5_, the ratios of the SRM transitions were the same, i.e., 1.4 and 1.0. Thus, there were no significant differences in fragment ion ratios between the stored and the processed samples. Moreover, the retention times were the same for both samples. It was concluded that it was nordiazepam-d_5_ that was detected in both the stored and processed samples.

Archontaki et al. [27] found that nordiazepam was transformed in acidic water solution, into the intermediate *N*-(2-benzoyl-4-chlorophenyl)-2-aminoacetamide with the molecular formula C_15_H_13_N_2_O_2_Cl and the monoisotopic mass 288.1 Da. The transformation product was crystalized and analyzed by LC-UV, GC-MS, ^1^H- and ^13^C-NMR, and IR spectroscopy. The chemical equilibrium of the intermediate and nordiazepam was reversible, but the further transformation of the intermediate to the final degradation product (C_13_H_10_NOCl) was however not reversible. In the present study, an ion with a retention time of 3.0 min and a mass-to-charge ratio of 289.0 was detected by LC-MS in a stored solution (pH 3.1) of nordiazepam. This ion might correspond to [M+H]^+^ of the transformation product of nordiazepam. Furthermore, the isotopic pattern for the ion at 3.0 min corresponded with the isotopic pattern for one chlorine atom. In addition, the chromatographic peak eluted before nordiazepam, which is in accordance with results from the separations of nordiazepam and *N*-(2-benzoyl-4-chlorophenyl)-2-aminoacetamide in the reversed phase system used by Archontaki et al. Thus, the detected peak in the stored acidic water solution, in this study, was most probably *N*-(2-benzoyl-4-chlorophenyl)-2-aminoacetamide. Moreover, the peak area ratio of nordiazepam to *N*-(2-benzoyl-4-chlorophenyl)-2-aminoacetamide was 0.75 (*n* = 2) in this stored water solution in the presented study. In the evaporated methanol mixtures (experimental details described in “Stability studies of diazepam and nordiazepam in acidic and neutral aqueous solutions”), the peak area ratio of nordiazepam to *N*-(2-benzoyl-4-chlorophenyl)-2-aminoacetamide however increased to 1.9 (6.8 RSD %, *n* = 4), i.e., the peak area of nordiazepam increased and the peak area of *N*-(2-benzoyl-4-chlorophenyl)-2-aminoacetamide decreased in comparison with the sample that was not evaporated. No peaks were detected for *N*-(2-benzoyl-4-chlorophenyl)-2-aminoacetamide or nordiazepam in the blank sample. These results strongly suggest that the chemical equilibrium of nordiazepam and the transformation product of nordiazepam, characterized by Archontaki et al. shifted from *N*-(2-benzoyl-4-chlorophenyl)-2-aminoacetamide to form nordiazepam during the evaporation of the SPE extracts. No peak was detected that could be correlated to the final degradation product (C_13_H_10_NOCl) of nordiazepam.

It was concluded that nordiazepam was readily transformed into *N*-(2-benzoyl-4-chlorophenyl)-2-aminoacetamide in the acidic water solution. Interestingly, nordiazepam was regenerated during the process of solid phase extraction. Thus, by the use of the stored solutions of nordiazepam at pH 3.0 as a reference in the calculations of extraction recoveries, the extraction recoveries are overestimated. These results are of importance during method validation, i.e., during assessments of storage conditions, extraction recoveries, and matrix effects. Moreover, the transformation of nordiazepam might influence the overall analytical results if an isotope-labeled analogue to nordiazepam is not used as an internal standard. It must also be emphasized that the transformation of nordiazepam might influence the accuracy of the method, not only during storage prior to solid phase extraction, but also depending on the pH of the solution used, e.g., reconstitution of the dried SPE extracts.

### Method validation

The developed method was validated by the use of the isotope-labeled analogues, diazepam-d_5_ and nordiazepam-d_5_ (“Method validation”), as these compounds were not detected in the environmental samples. The advantage by using the labeled analogues for the method validation is that the method can be validated at trace levels in the actual matrix in which the analytes are quantified [6, 7]. The *relative extraction recoveries* in the treated wastewater samples were ≥ 87% for diazepam-d_5_ and nordiazepam-d_5_ at the high and low concentration (Table 3). The obtained values are in the ranges of what can be expected when trace-level concentrations of pharmaceuticals are extracted from complex matrices [34, 35]. The *absolute extraction recoveries* were lower, 63–86%, as the analytes were subjected to ion suppression (Table 3). At the low concentration, the *matrix effects* (ME %) were 76 ± 14% and 88 ± 14% for diazepam-d_5_ and nordiazepam-d_5_, respectively (Table 3). At the high concentration, the effect of the matrix and the RSD values were in the same range as at the low concentration. These figures of ME % are within the acceptable range, as results from other studies show that the matrix effect obtained with environmental water matrices can be relatively high [10]. The *accuracy* of the method was determined by the determination of the SPE recoveries at the low and the high concentration of diazepam-d_5_ and nordiazepam-d_5_ (Table 3). The relative recoveries were 88 ± 7.6% and 87 ± 12% for diazepam-d_5_ at the low and high concentration, respectively, and 98 ± 7.8% and 99 ± 6.1% for nordiazepam-d_5_.

**Table 3:** The relative and absolute recoveries, matrix effects, limit of quantification, and limit of detection for diazepam-d_5_ and nordiazepam-d_5_ in treated wastewater. The recoveries (%) and matrix effects (%) determined for the developed method at a sample pH of 7 at one low and one concentration of diazepam-d_5_ and nordiazepam-d_5_. For the LOQ, the average signal-to-noise ratios (*n* = 3) and the RSD values (*n* = 3) are given. For the LOQ, the signal-to-noise ratios (*n* = 1) are given. The experimental details are described in “Method validation”

Compound	Concentration (pM), *n =* 5	Relative recovery ± RSD %, *n =* 5	Absolute recovery ± RSD %, *n =* 5	Matrix effect ± RSD %, *n =* 5
Diazepam-d_5_	50	88 ± 7.6	67 ± 7.6	76 ± 14
Nordiazepam-d_5_	50	98 ± 7.8	86 ± 7.8	88 ± 11
Diazepam-d_5_	250	87 ± 12	63 ± 12	72 ± 4.6
Nordiazepam-d_5_	250	99 ± 6.1	85 ± 6.1	80 ± 9.8
	LOQ (pM)	S/N *n* = 3	RSD % *n* = 3	LOD (S/N) (pM), *n =* 1
Diazepam-d_5_	5.0	11.7	12.7	1.7 (3.7)
Nordiazepam-d_5_	5.0	10.4	15.9	2.0 (3.6)

The precision of the chromatographic system, expressed as the RSD values of the retention times obtained for diazepam-d_5_ and nordiazepam-d_5_ in extracted wastewater samples, was ≤ 0.62%. The RSD values of the peak areas for diazepam-d_5_ and nordiazepam-d_5_ in the extracted wastewater samples were ≤ 7.8% (“The liquid chromatography tandem quadrupole mass spectrometry systems”). Furthermore, the linearity, expressed as the correlation coefficient (*R*^2^) of the calibration curves in the treated wastewater samples, was 0.988 and 0.957 for diazepam and nordiazepam, respectively.

No carryover in the LC-MS/MS system was observed in this study as no peaks of the analytes or the isotope-labeled compounds were detected in any of the injected purified Millipore water samples. There was no indication that any cross-contamination occurred during the sample handling or the solid phase extraction as the extracted phosphate buffer samples did not contain any of the target compounds. The risk of obtaining false positive findings as a result of self-contamination was therefore considered to have been minimized in this study.

The LOQ and LOD for diazepam-d_5_ and nordiazepam-d_5_ were determined in treated wastewater samples. The limits of quantification were set to 5.0 pM (1.4 ng L^−1^) for both diazepam-d_5_ and nordiazepam-d_5_ where the signal-to-noise ratios were about 10 and the obtained precision were 12.7 and 15.9 RSD % (*n* = 3) for the respective compounds (Table 3), i.e., within the stipulated precision of 20% [31]. The obtained LOQ values in the present study are in the range of what has been achieved in other studies for diazepam and nordiazepam in treated wastewater samples [34]. However, in that study, a volume of 200 mL treated wastewater was extracted [34], in comparison with 75 mL in our presented method. The limits of detection were 1.7 pM (0.49 ng L^−1^) and 2.0 pM (0.55 ng L^−1^) for diazepam-d_5_ and nordiazepam-d_5_, respectively (Table 3).

As discussed above (“The stability of diazepam and nordiazepam in acidic and neutral aqueous samples,” Fig. 2), diazepam and nordiazepam were shown to be stable for 12 days at a sample pH of 7.0 when stored at room temperature or at 4 °C.

### Quantification of diazepam and nordiazepam in environmental water samples

The developed LC-MS/MS method was applied to environmental water samples for the determination of diazepam and nordiazepam. It must be emphasized that the developed method can be employed for the determinations of diazepam and nordiazepam in environmental samples in acidic conditions if ideal internal standards are added to the samples before storage, i.e., isotope-labeled compounds of the target compounds. In this study, the isotope-labeled compounds, diazepam-d_5_ and nordiazepam-d_5,_ were used as internal standards to compensate for the potential transformation of the compounds and other losses as well as variations during the analysis.

Treated wastewater and surface water samples were analyzed for diazepam and nordiazepam. There were no significant differences, at the 5% level in a *t* test, between the ion ratios obtained for standard solutions (Table 1) and the ones obtained for the “naturally” occurring diazepam or nordiazepam (*P* ≥ 0.07). The concentrations of diazepam and nordiazepam were determined to be 8.5 (2.4 ng L^−1^) and 66 pM (18 ng L^−1^), respectively. In samples from the same wastewater treatment plant collected 14 days later, the concentrations were determined to be 7.5 (2.1 ng L^−1^) and 75 pM (20 ng L^−1^) for diazepam and nordiazepam, respectively. Thus, the concentrations of nordiazepam were determined to be about one order of magnitude higher than the ones for diazepam. These results are in accordance with results from other studies of wastewater effluents [12]. Moreover, in some treated wastewater samples, reported in the literature, nordiazepam was quantified, but diazepam was not detected [14, 15]. In the present study, neither diazepam nor nordiazepam was detected in surface water collected from the River Fyris, 3 km upstream from the wastewater treatment plant Kungsängsverket, indicating few anthropogenic wastewater emissions upstream.

## Conclusions

This study highlights the importance of using a suitable blank matrix, reference standards, and sample storage conditions in the validation process as well as for further routine work in order to obtain accurate measurements. Nordiazepam was shown to be stable at a sample pH of 7 for 12 days; the compound was however unstable in the aqueous solution at a sample pH of 3. The transformed nordiazepam at pH 3 was regenerated to nordiazepam in the process of solid phase extraction. This chemical equilibrium between nordiazepam and the transformed product might influence the overall accuracy of the LC-MS/MS method if the wastewater samples are stored at low sample pH prior to analysis. In the presented study, the initial extraction recoveries were overestimated for nordiazepam due to the chemical equilibrium in samples at low pH, i.e., we demonstrate that the chemical equilibrium between two compounds can affect the method validation.

The developed LC-MS/MS method was validated in wastewater effluents by the use of the isotope-labeled standards of diazepam and nordiazepam. Diazepam and nordiazepam were quantified in treated wastewater and the concentration of nordiazepam was determined to be one order of magnitude higher than the concentration of diazepam.
